# Incremental Peritoneal Dialysis May be Beneficial for Preserving Residual Renal Function Compared to Full-dose Peritoneal Dialysis

**DOI:** 10.1038/s41598-019-46654-2

**Published:** 2019-07-12

**Authors:** Yeonhee Lee, Sung Won Chung, Seokwoo Park, Hyunjin Ryu, Hajeong Lee, Dong Ki Kim, Kwon Wook Joo, Curie Ahn, Joongyub Lee, Kook-Hwan Oh

**Affiliations:** 1 Department of Internal Medicine, Seoul National University College of Medicine, Seoul, Korea; 2 Prevention and Management Center, Inha University Hospital, Incheon, Korea

**Keywords:** End-stage renal disease, Peritoneal dialysis

## Abstract

Maintaining residual renal function (RRF) is a crucial issue in peritoneal dialysis (PD). Incremental dialysis is the practice of initiating PD exchanges less than four times a day in consideration of RRF, and increasing dialysis dose in a step-wise manner as the RRF decreases. We aimed to compare the outcomes of incremental PD and full-dose PD in terms of RRF preservation and other outcomes. This was a single-center, observational study. Data were extracted retrospectively from a cohort of incident PD patients over 16 years old who started PD between 2007 and 2015 in the PD Unit of Seoul National University Hospital. We used inverse probability weighting (IPW) adjustment based on propensity scores to balance covariates between the incremental and full-dose PD groups. Multivariate, time-dependent Cox analyses were performed. Among 347 incident PD patients, 176 underwent incremental PD and 171 underwent conventional full-dose PD. After IPW adjustment, the incremental PD group exhibited a lower risk of developing anuria (hazard ratio [HR] 0.61, 95% confidence interval [CI] 0.43–0.88). Patient survival, technique survival, and peritonitis-free survival were all similar between these groups (*P* > 0.05 by log-rank test). Incremental PD was beneficial for preserving RRF and showed similar patient survival when compared to conventional full-dose PD.

## Introduction

Maintaining residual renal function (RRF) is a crucial issue in patients with end-stage renal disease (ESRD), because it has been reported in numerous observational studies to play an important role in the adequacy of dialysis, quality of life, and patient survival in dialysis patients^1–7^. A higher RRF is associated with better patient survival in both peritoneal dialysis (PD) and hemodialysis (HD) patients^8,9^. PD is the preferred renal replacement modality for the preservation of RRF^10^; in particular, the rate of RRF decline was slower in patients receiving PD than in HD patients^11,12^. Moreover, PD can slow the decline of RRF compared to the natural slope of RRF decline prior to dialysis initiation^13,14^.

Incremental PD is the practice of initiating PD exchanges less than four times a day in consideration of RRF, and increasing dialysis dose in a step-wise manner as the RRF decreases, by achieving a minimum weekly Kt/V target of 1.7 as suggested by the NKF-KDOQI (National Kidney Foundation – Kidney Disease Outcomes Quality Initiative)^15^ and ISPD (International society for peritoneal dialysis) 2006 Guidelines^16^ and not falling below the acceptable minimum. This gradual initiation of dialysis may make the process of dialysis less invasive and better tolerated hemodynamically, as well as allowing for a greater adaptation to patients^17,18^.

Continuous ambulatory peritoneal dialysis (CAPD) may be an ideal method for beginning an incremental dialysis strategy. Starting dialysis gradually, with 1 to 2 manual exchanges a day and graduating up to a maximal 4 exchanges a day (full-dose) if needed, could be an acceptable method to more gently transition into renal replacement therapy, and is better tolerated by patients in terms of lifestyle disruption.

Not only did more frequent and longer dialysis not improve clinical outcomes in either PD or HD patients, randomized controlled trials have also shown inconsistent results^19–22^. Indeed, more frequent and lengthy dialysis may even accelerate RRF decline^23^. Moreover, previous studies have suggested that an incremental approach to PD initiation has some advantages associated with the lower number of exchanges per session, such as a lower incidence of peritonitis, a better quality of life, reduced cost, and preservation of RRF^17,24,25^. However, effects of incremental PD on the RRF and technique survival have not been widely studied yet. The objective of this study was to compare the outcomes of incremental PD and full-dose PD in terms of RRF preservation and other PD-related outcomes.

## Materials and Methods

### Study design and patients

This was a single-center, retrospective cohort study. Data were extracted retrospectively from a cohort of incident PD patients who commenced PD between January 1, 2007 and December 31, 2015 in the PD Unit of Seoul National University Hospital. Patients were excluded from the study if they met any of the following criteria: total duration of PD less than 6 months, initiation of PD at another hospital, urine volume of < 200 mL per day at the time of initiating PD, previous hemodialysis, less than 16 years of age, and incomplete study data. Patients who continued PD were followed until July 31, 2017 and were censored at the time of death or loss to follow-up. To control for baseline comorbidities in the analysis, patient demographics, clinical, and laboratory data were recorded.

The RRF was assessed by GFR calculated as the mean of urea and creatinine clearances normalized to 1.73 m^2^ BSA calculated using the du Bois and du Bois formula, as follows: 0.007184 (body weight in kg)^0.425^ (height in cm)^0.725^. Every 6 months, including the first month after dialysis, a total weekly Kt/V was calculated relating the values taken from the 24-hour collection of urine and PD fluid to assess peritoneal and renal solute clearance was.

Operationally, full-dose PD was defined as the initiation of PD with 3 or more exchanges per day for CAPD, 7 days a week, irrespective of RRF. Incremental PD was defined as one or two dwells per day on CAPD, 7 days a week, and a peritoneal Kt/V < 1.7 per week, but a total Kt/V ≥ 1.7 per week^26–28^. However, since no patients treated with APD met the stringent criteria of incremental APD^28^, we analyzed data after excluding all the patients treated with APD. Also, patients who did not comply with our operational definition of full dose or incremental PD were excluded from the study. The comparison between incremental and full-dose PD approaches was based on an intention-to-treat analysis. That is, for the incremental PD group, the delivered PD dose was gradually increased to a full-dose over time, as some patients’ RRF decreased. In this case, such patients were categorized as the incremental PD group.

This study was approved by the Seoul National University Hospital Institutional Review Board (No. H-1706-114-860) and complied with the Declaration of Helsinki. The informed consents were waived by the IRB, because this study was a retrospective study.

### Outcomes

The primary outcome of this study was time to anuria from initiation of dialysis, such that the urine volume reflected the RRF. Anuria was defined as a urine volume of < 100 mL per day.

Secondary outcomes were peritonitis, technique failure, and all-cause mortality. Peritonitis was diagnosed according to the 2006 guidelines from the ISPD^29^, and peritonitis rate was calculated as the number of peritonitis episodes per patient-year at risk. PD technique failure was defined as a transfer to hemodialysis and death directly related to PD-related complication. Causes of PD technique failure were as follows: peritonitis, inadequate PD, catheter complications, other abdominal or PD-related complications, and psychosocial barriers^30,31^. For analysis of time-to-technique failure, patients were censored at the time of death due to other causes, kidney transplantation, and follow-up loss. Death was determined using either hospital medical records or data from the National Database of Statistics Korea by using the Korean resident registration number.

### Adjustment for differences between groups

Given that our study used non-randomized, observational data, it was anticipated that the incremental and full-dose PD groups would differ substantially with respect to characteristics at the point of dialysis initiation, because the patient’s RRF may influence the selection between the two methods. The propensity score to estimate the probability, on the basis of patient characteristics, that patients would be selected for incremental PD was calculated with the use of logistic regression analyses to balance the baseline characteristics of the patients’ between-group differences. Variables included in the propensity score calculation are shown in Table 1. Then, IPW based on the propensity score was used as a tool for creating balance, with weighting each patient who underwent full-dose PD by the inverse of the probability that he or she would be selected for full-dose PD and weighting each patient who underwent incremental PD by the inverse of the probability that he or she would be selected for incremental PD. We verified the performance of the IPW procedure by comparing the distribution of covariates and standardized differences between groups both before and after IPW.

**Table 1:** Baseline characteristics for the cohort before and after inverse probability weighting (IPW) adjustment.

	Unadjusted (without IPW)				After IPW adjustment			
	Incremental PD (n = 176)	Full-dose PD (n = 171)	*P* value	Standardized Difference	Incremental PD (n = 176)	Full-dose PD (n = 171)	*P* value	Standardized Difference
Age (yr)	49.2 ± 12.63	43.9 ± 13.07	<0.001	0.41	49.2 ± 12.63	48.2 ± 13.66	0.666	0.08
Male sex (%)	52.3	52.0	1.000	<0.01	52.3	50.3	0.811	0.04
BMI (kg/m^2^)	22.3 ± 3.04	22.4 ± 3.37	0.699	0.04	22.3 ± 3.04	22.6 ± 2.90	0.530	0.10
Primary renal disease (%)			0.306	0.29			0.613	0.32
Diabetic nephropathy	29.0	19.3		0.22	29.0	29.0		0.00
Hypertension	14.8	21.6		0.17	14.8	21.3		0.16
Glomerulonephritis	33.0	38.0		0.10	33.0	27.6		0.11
Polycystic kidney disease	2.8	1.8		0.06	2.8	1.7		0.07
Graft failure	6.2	4.7		0.06	6.2	5.0		0.05
Others	5.1	4.7		0.01	5.1	10.4		0.19
Unknown	9.1	9.9		0.02	9.1	4.9		0.16
Comorbidities (%)								
Hypertension	88.1	84.8	0.463	0.09	88.1	89.0	0.839	0.03
Diabetes mellitus	33.0	24.0	0.083	0.20	33.0	30.5	0.763	0.05
Visual disturbance	19.9	12.3	0.075	0.20	19.9	20.8	0.908	0.02
Ischemic heart disease	4.5	1.8	0.239	0.16	4.5	2.3	0.414	0.12
Cerebrovascular disease	1.7	2.3	0.969	0.04	1.7	0.9	0.444	0.06
Peripheral vascular disease	5.1	2.9	0.445	0.11	5.1	1.8	0.093	0.18
Congestive heart failure	7.4	3.5	0.177	0.17	7.4	2.4	0.063	0.23
Hepatitis	6.2	5.3	0.870	0.04	6.2	10.5	0.400	0.15
Malignancy	3.4	2.3	0.784	0.06	3.4	2.6	0.720	0.04
Kidney transplantation	8.0	4.7	0.302	0.13	8.0	5.0	0.485	0.12
Medication (%)								
ISA	4.5	5.3	0.951	0.03	4.5	4.8	0.936	0.01
ACEI/ARB	79.0	72.5	0.201	0.15	79.0	70.2	0.211	0.20
Davies comorbidity index			0.04	0.27			0.544	0.13
0 (Low risk)	52.8	65.5		0.26	52.8	59.2		0.12
1–2 (Medium risk)	46.0	32.7		0.27	46.0	40.2		0.11
≥3 (High risk)	1.1	1.8		0.05	1.1	0.6		0.05
Urine volume (mL/day)	1576 ± 595.2	943 ± 530.1	<0.001	1.12	1576 ± 595.2	1451 ± 494.8	0.111	0.22
Residual renal function (mL/min/1.73 m^2^)	6.9 ± 3.00	4.2 ± 2.36	<0.001	1.00	6.9 ± 3.00	6.5 ± 2.43	0.298	0.16
Weekly peritoneal Kt/V	1.05 ± 0.48	1.48 ± 0.350	<0.001	1.01	1.05 ± 0.48	1.27 ± 0.36	<0.001	0.52
Weekly renal Kt/V	1.38 ± 0.64	0.74 ± 0.449	<0.001	1.15	1.38 ± 0.64	1.22 ± 0.51	0.099	0.27
Total weekly Kt/V	2.42 ± 0.68	2.22 ± 0.531	0.002	0.33	2.42 ± 0.68	2.49 ± 0.59	0.515	0.10
Peritoneal CrCl (L/week/1.73 m^2^)	26.1 ± 13.06	38.0 ± 9.68	<0.001	1.03	26.1 ± 13.06	33.9 ± 9.54	<0.001	0.68
Total CrCl (L/week/1.73 m^2^)	96.1 ± 29.24	80.7 ± 24.31	<0.001	0.57	96.1 ± 29.24	99.4 ± 25.25	0.451	0.12

Abbreviations: PD, peritoneal dialysis; BMI, body mass index; ISA, immunosuppressive agent; ACEI, angiotensin-converting enzyme inhibitor; ARB, angiotensin II receptor blocker; CrCl, creatinine clearance.

Values are expressed as mean ± SD or percentages.

The propensity score was calculated using a logistic regression model, regressed on observed baseline characteristics (age, sex, BMI, primary renal disease, all comorbidities, history of medication such as immunosuppressive agent and ACEI or ARB, urine volume, weekly renal Kt/V, except for the variables such as residual renal function, weekly peritoneal Kt/V, total weekly Kt/V and creatinine clearance, with which multicollinearity was detected.

Peritoneal and renal solute clearances were measured at time of first month after dialysis.

### Statistical analysis

The data are described with means ± standard deviations for continuous variables, and frequencies and proportions for categorical variables. A comparison of baseline characteristics was performed with the Wilcoxon rank-sum test for continuous variables, and the use of the Pearson chi-square test for categorical variables. We estimated Kaplan-Meier survival curves adjusted with the use of inverse probability weighting, and compared anuria-free survival between groups using the log-rank test. Since the proportional hazards assumption was not met for the incremental group variable as shown by the crossing survival curves, multivariable, time-dependent Cox proportional hazards regression analyses were performed to identify the association between incremental approach of PD and survival. We then calculated an adjusted hazard ratio and created weighted adjusted survival curves. All tests were two-sided, and *P* < 0.05 was considered statistically significant. The analyses were performed with the use of the statistical software packages SPSS (version 22) and R (version 3.5.0).

## Results

### Population characteristics

**Figure 1:**
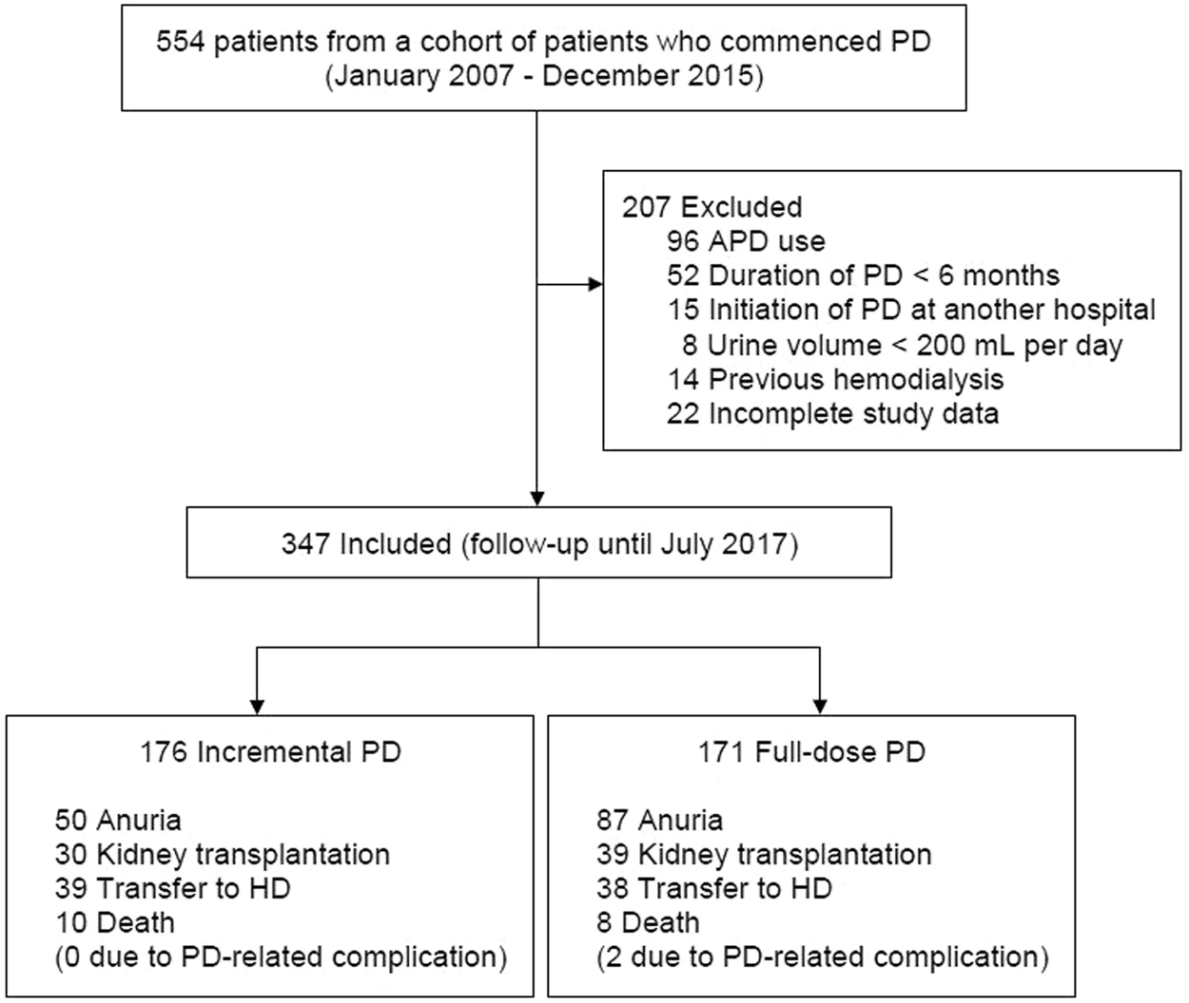
Flow of patients in the cohort. PD, peritoneal dialysis; HD, hemodialysis.

A total of 347 incident PD patients, assessed were eligible for inclusion, started PD in the PD Unit of Seoul National University Hospital during the study period (January 1, 2007 to December 31, 2015); 176 underwent incremental PD and 171 underwent conventional full-dose PD (Fig. 1). The baseline characteristics of the patients are shown in Table 1. Before adjustment using inverse probability weighting (IPW), patients in the incremental PD group were generally older, and a higher proportion were medium-risk patients by the Davies comorbidity index, compared with patients who initiated full-dose PD. There was no significant difference in sex, the composition of primary renal disease, comorbidities, and the use of ACEI or ARB between the two groups. The baseline mean urine volume in each group were 1576 ± 595.2 mL in the incremental PD group and 943 ± 530.1 mL in the full-dose PD group. Peritoneal and renal solute clearances were measured between four and six weeks after initiating PD. Compared with patients initiating full-dose PD, the incremental PD group had a higher residual renal function, weekly renal Kt/V and urine volume, whereas had a lower peritoneal Kt/V and peritoneal CrCl, than the full-dose PD group. For the incremental PD group, as patients’ RRF decreased, the delivered PD dose gradually increased over time. The median duration of incremental PD was 2.6 years (interquartile range [IQR] 1.6–4.5 years, maximum 9.2 years). After IPW adjustment, the balance in baseline characteristics, especially the variables of renal solute clearances and urine volume between incremental and full-dose PD groups improved (Table 1).

### Residual renal function – anuria-free survival

**Figure 2:**
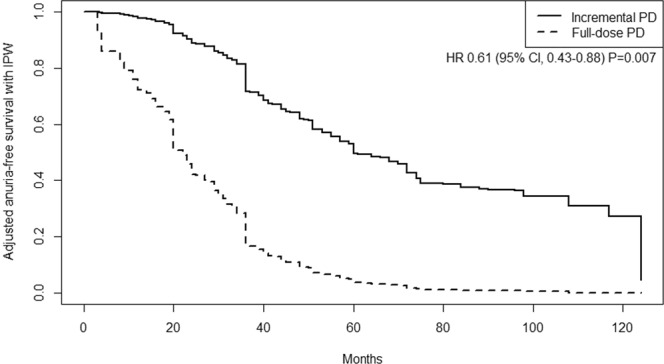
Inversed probability weighted, adjusted anuria-free survival using time-dependent Cox proportional hazards model.

Fifty (28.4%) patients in the incremental PD group and 87 (50.9%) in the full-dose PD group developed anuria over time. The median follow-up duration of all patients was 5.9 years (IQR 3.3–7.8 years, maximum 10.6 years). Figure 2 shows the cumulative probability of remaining anuria-free in two groups. Incremental PD was associated with significantly lower risks of anuria (hazard ratio [HR] 0.61, 95% confidence interval [CI] 0.43–0.88; *P* = 0.007) in univariable, time-dependent Cox proportional hazards model analyses with IPW-weighted for anuria (Fig. 2). In multivariable analyses, the other independent predictors of developing anuria were younger age and history of kidney transplantation (Table 2). There was no significant association between initial urine volume and anuria.

**Table 2:** Results of multivariable, time-dependent Cox proportional hazards model analyses with IPW-weighted for anuria.

Variable	HR (95% CI)	*P* value
Incremental PD	0.55 (0.34–0.89)	0.015
Age	0.97 (0.95–0.98)	<0.001
Kidney transplantation	7.45 (1.00–55.61)	0.05
Urine volume (per 100 mL)	0.97 (0.94–1.01)	0.162

Abbreviations: IPW, inverse probability weighting; HR, hazard ratio; CI, confidence interval; PD, peritoneal dialysis.

### Incidence of peritonitis and peritonitis-free survival

Both the time to the first episode of peritonitis and the incidence of peritonitis were assessed. The analysis of time-to-first peritonitis included 144 episodes of peritonitis over 1,400 patient-years. The incidence rates of first peritonitis were 0.10 episodes per patient-year (95% CI 0.08–0.13) in the incremental PD group and 0.10 per patient-year (95% CI 0.08–0.12) in the full-dose PD group (Table 3). The overall median time to the first peritonitis was 2.3 years. A Kaplan-Meier curve for the probability of remaining peritonitis-free showed no difference between the two groups (*P* = 0.860 by log-rank test).

**Table 3:** Incidence of first peritonitis (144 episodes) among 347 study participants.

	First episode	
	Incremental PD (n = 176)	Full-dose PD (n = 171)
Number of first peritonitis	71	73
Follow-up time (patient-year)	692.9	750.9
Peritonitis incidence (episode / patient-year) (95% CI)	0.10 (0.08–0.13)	0.10 (0.08–0.12)

Abbreviations: PD, peritoneal dialysis; CI, confidence interval.

### Technique survival and mortality

A total of 78 (22.5%) patients experienced PD technique failure over the follow-up period; of them, 2 patients died due to PD-related complications. When we compare the technique failure-free survival between the incremental and full-dose PD groups using the Kaplan-Meier method, it was not significantly different (The median time to the technique failure 2.7 years in the incremental PD group vs. 2.9 years in the full-dose PD group; *P* = 0.332 by log-rank test).

**Figure 3:**
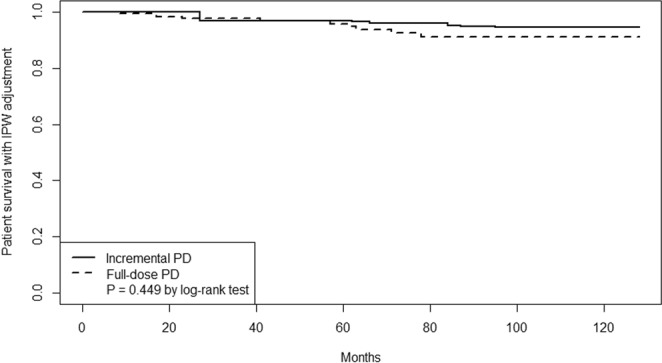
Kaplan-Meier survival estimates using inverse probability weighting (IPW).

Death from any cause occurred in 10 patients in the incremental PD group (10.9 events per 1000 person-years; 95% CI 5.2–20.0) and 8 in the full-dose PD group (7.6 events per 1000 person-years; 95% CI 3.3–15.0). In the log-rank test, the between-group difference was not significant (*P* = 0.449) (Fig. 3).

## Discussion

The basic assumption of incremental dialysis is to reach the minimal target for adequate dialysis by summing renal function and dialysis dose. Mehrotra *et al*. and Golper first described an early and incremental approach to PD^32,33^. Early studies included only a small number of patients, and lacked control groups and any statistical comparison. De Vecchi *et al*. first reported working activity, degree of rehabilitation, and quality of life in incremental PD patients; quality of life and social rehabilitation were better preserved.

There is still disagreement on whether PD should begin with a full dose or with incremental doses to compensate for the amount of Kt/V no longer supplied by the RRF. Despite this consideration, incremental PD has become increasingly relevant with a world-wide trend to gradually initiate chronic dialysis at higher levels of renal function over the past 2 decades; in Italy, 29% of patients start PD with an incremental approach, and 51% of patients were on incremental PD at a single Canadian academic center^17,34^. Moreover, it is referenced as an option in a number of prominent clinical guidelines and practice recommendations. The possible benefits of incremental PD, including an improved quality of life, reduced glucose exposure to the peritoneal membrane, better peritonitis-free survival, longer preservation of RRF, and lower costs, may explain its widespread use. It is also considered an ideal bridge to renal transplantation.

This study showed that incremental PD was beneficial for preserving RRF (as demonstrated by a longer anuria-free time) compared to conventional full-dose PD, with similar technique survival, peritonitis-free survival, and mortality. The results of the study give support to the preserving effect of incremental PD on the course of RRF. Golper and Mehrotra recently expanded on Bricker’s intact nephron hypothesis, and suggested that an incremental approach to the initiation of dialysis might help preserve RRF by both reducing nephron hyperfiltration and deactivating certain adaptive stimuli which occur in the setting of reduced nephron numbers^35^. Moreover, they noted that an incremental approach to the transitioning of patients from CKD to dialysis may make the patients more receptive to treatment, as it would provide a better quality of life without interfering with their daily activities.

Our findings confirm previous studies that younger age is associated with a rapid decrease in RRF^1^. In particular, patients initiating PD after kidney transplant failure also suffered a relatively rapid loss in RRF^36,37^, probably as a consequence of cessation of immunosuppression and associated inflammation^37^.

The strengths of this study include its relatively large cohort and inclusiveness. We included all incident CAPD patients during the study period in our PD unit. Developing a large-scale, multi-centre, randomized, controlled trials comparing incremental PD with full-dose PD is not feasible. Instead, we were able to obtain stable and reliable clinical data from a relatively large number of PD patients with regular monitoring of RRF and dialysis adequacy. IPW maximized data available while maintaining balance of measured covariates between groups and producing a minimally biased effect estimate^38^.

This study has several limitations. We acknowledge the limitations inherent to single-center experience. The study is an observational study, and the unadjusted clinical profile and propensity scores for incremental PD differed between the treatment groups. The clinician’s decision between full-dose and incremental PD might be affected by a number of clinical factors such as RRF, lifestyle, body size, gender, and comorbidities. Subjects treated with APD, which accounted for 20% of total PD population were not included in the analysis, since no APD treatment in our center met the stringent criteria of incremental APD. Although we excluded patients with urine volume of < 200 mL per day and tried to adjust all measurable risk factors using IPW for achieving a good balance between the incremental and full-dose PD groups, the potential remains for unmeasured confounders to have influenced the findings. Also, a competing risk analysis was not conducted. Some patients died before anuria events, although these cases were appropriately censored. Lastly, the specific prescriptions of incremental PD, mainly based on the summation of peritoneal and renal small solute clearances, were inconsistent and diverse. Dialysis regimens for incremental approach have only been established in a few studies, and their successful implemental requires more clinical experience.

In conclusion, this study demonstrates that incremental PD was beneficial for preserving residual renal function (RRF) compared to conventional full-dose PD, with similar technique survival and mortality rates between the two groups. Therefore, incremental PD is a safe modality for initiating dialysis, and a longer preservation of RRF may have additional positive effect on patients who are waiting for kidney transplantation. Further prospective studies to explore the effects of incremental PD on RRF are needed.
